# Human-Induced Pluripotent Stem Cells in Plastic and Reconstructive Surgery

**DOI:** 10.3390/ijms25031863

**Published:** 2024-02-03

**Authors:** Nina Hadzimustafic, Andrew D’Elia, Valentina Shamoun, Siba Haykal

**Affiliations:** 1 Temerty Faculty of Medicine, University of Toronto, Toronto, ON M5S 1A8, Canada; nina.hadzimustafic@mail.utoronto.ca (N.H.); andrew.delia@mail.utoronto.ca (A.D.); valentina.shamoun@mail.utoronto.ca (V.S.); 2 Department of Plastic and Reconstructive Surgery, University Health Network, Toronto, ON M5G 2C4, Canada

**Keywords:** induced pluripotent stem cells (iPSC), plastic surgery, regenerative medicine, tissue engineering, reconstructive surgery

## Abstract

A hallmark of plastic and reconstructive surgery is restoring form and function. Historically, tissue procured from healthy portions of a patient’s body has been used to fill defects, but this is limited by tissue availability. Human-induced pluripotent stem cells (hiPSCs) are stem cells derived from the de-differentiation of mature somatic cells. hiPSCs are of particular interest in plastic surgery as they have the capacity to be re-differentiated into more mature cells, and cultured to grow tissues. This review aims to evaluate the applications of hiPSCs in the plastic surgery context, with a focus on recent advances and limitations. The use of hiPSCs and non-human iPSCs has been researched in the context of skin, nerve, vasculature, skeletal muscle, cartilage, and bone regeneration. hiPSCs offer a future for regenerated autologous skin grafts, flaps comprised of various tissue types, and whole functional units such as the face and limbs. Also, they can be used to model diseases affecting tissues of interest in plastic surgery, such as skin cancers, epidermolysis bullosa, and scleroderma. Tumorigenicity, immunogenicity and pragmatism still pose significant limitations. Further research is required to identify appropriate somatic origin and induction techniques to harness the epigenetic memory of hiPSCs or identify methods to manipulate epigenetic memory.

## 1. Introduction

A core objective of regenerative medicine is to restore bodily form and function in the context of tissue abnormality or insult [1,2]. In plastic and reconstructive surgery, tissue derived from a healthy portion of the patient’s body is repurposed to cover, fill, and recapitulate the function of tissue defects [3,4]. Tissue defects range from acute and chronic wounds and large soft tissue defects to total structure loss, such as of the face and limb [3,5]. Identifying a viable and suitable tissue source remains a significant limitation in plastic surgery practice [6,7,8]. Current reconstructive methods hinge on the use of either autologous or allogeneic donor tissue, engineered grafts or alloplastic implants [9,10,11]. This can present a particularly challenging barrier in the context of widespread injuries wherein donor site availability becomes quite limited.

A growing body of research has introduced stem cells as a promising new approach to disease modeling, drug screening, and regenerative medicine. They are of particular interest to plastic surgeons as a novel source of additional tissue for use in reconstruction [12]. Stem cells are unique from other cell types in their ability to differentiate into a number of possible phenotypes. The process of cellular differentiation is one which results in the progressive specialization and restricted developmental potential of cells [13]. Stem cells are capable of both self-renewal and differentiation into more mature cell types [14]. They can be categorized as totipotent, giving rise to all embryonic and adult lineage cells (found within the first four cleavages of an embryo); pluripotent, giving rise to all adult lineage cells; and multipotent, giving rise to a subset of cells within a lineage [15].

Pluripotent stem cells (PSCs) have become of the highest interest for scientific advances. PSCs are further broken down into embryonic stem cells (ESCs) and induced pluripotent stem cells (iPSCs). ESCs are derived from the inner cell mass of a blastocyst, whilst iPSCs are derived from the de-differentiation of somatic cells. Somatic cells are reprogrammed into pluripotency by expressing four transcription factors: Oct4/Sox2/c-Myc/KLF4 or Oct4/Sox2/NANOG/LIN28 [16,17,18]. Historically, PSCs have been hypothesized to present significant advantages to researchers because they offer the possibility of patient-specific cells whilst circumventing the tumorigenicity [19] and immunocompatibility [20] concerns attached to ESCs [21]. Ethical considerations regarding the derivation of stem cells also preclude the clinical translation of stem cells in reconstructive surgery. In the future, iPSCs could offer an ethically sound alternative to ESCs while maintaining the promise of a patient-specific, personalized approach to tissue regeneration.

Multipotent stem cells have also been used in disease modeling, drug screening, and a variety of clinical contexts, demonstrating success in the regeneration of skin, soft tissue, bone, cartilage, and nerves. Particularly notable is the use of adipose-derived stem cells (ADSCs), which have been lauded for their ease of procurement, culture, multipotency, viability and potent expression of various mitogens, cytokines, and pro-healing agents. They are argued to be vital to the neovascularization and survival of tissues post engraftment [22,23]. Mesenchymal stem cells (MSCs) are another multipotent cell line that has been explored for similar purposes. Clinical trials with MSCs cite their use as a source of immunomodulatory and trophic factors that can cultivate regenerative microenvironments [24,25,26]. Interestingly, MSCs can be derived from autologous or allogeneic sources, and coordinate tissue progenitor cell function to expedite healing in damaged tissues [27]. Our focus is on iPSCs due to the specific advantages they offer in the research setting over ADSCs and MSCs. ADSCs present laboratory challenges such as the availability of donor tissue and knowledge of the exact historical culture conditions required to maintain the culture; meanwhile, iPSCs can be established as a cell line and can be repeatedly differentiated for replicates in research [28]. MSCs can only be differentiated into mesodermal tissues, unlike iPSCs, which can be differentiated into endodermal, mesodermal, and ectodermal tissues, covering the majority of tissue models required for plastic surgery research, including skin [29,30,31].

The inception of iPSCs represents a substantial milestone in regenerative medicine. Herein, we explore advances in iPSC-based techniques as they pertain to plastic surgery and eventual translation to human-induced pluripotent stem cell (hiPSC) work. New approaches to disease modeling and drug testing [32], the enhancement of wound healing [33,34,35], the regeneration of soft tissue [12] and, most ambitiously, the potential to grow vascularized composite tissues [36,37] such as limbs will be discussed. Although current limitations and challenges such as iPSC-associated tumorigenicity, immunogenicity and pragmatism stand to humble these advancements, the outlook on iPSC-derived therapies is bright, and will one day redefine the scope and capabilities of plastic surgery.

## 2. Methods

A comprehensive literature search using Google Scholar, OVID and PubMed was performed. The final search was completed in November 2023. The full search strategy comprised the terms “hiPSC” OR “iPSC” AND “plastic surgery” OR “reconstructive surgery” OR “tissue engineering” OR “regenerative medicine”. An English language filter was applied. Each database was searched from inception until November 2023. Search results were independently reviewed by three authors, NH, AD, and VS. Non-English journal articles were excluded from the review.

## 3. Applications of hiPSCs in Reconstructive Surgery

Section 3 will discuss the current landscape of hiPSC use within plastic and reconstructive surgery, including wound-healing and disease-modelling applications. It will also discuss advances in the regeneration of skin, vasculature, nerves, skeletal muscle, cartilage, and bone, as well as the strides made in regenerative efforts to achieve vascularized composite allotransplantation. The most exciting potential clinical application of hiPSCs is that of custom-grown tissue to help restore various deformities that otherwise require biomaterials often complicated by infection, fibrosis, contracture, or tissue with limited donor sites [12]. iPSC development provides new hope for patients with burns or large skin defects, as their treatment is are often limited by donor site availability and the quality of wound healing [38,39]. Fu et al. harvested human skin fibroblasts from burn patients and successfully generated patient-specific iPSCs, providing an experimental basis for this potential future clinical application [40]. Another potential future application of iPSCs in plastic surgery is skin rejuvenation. A study by Bakhshandeh et al. showed that iPSCs can release microvesicles, which, when applied to dermal fibroblasts, upregulate collagen expression for skin regeneration [41]. Another important hypothesized clinical application of iPSCs is the treatment of peripheral neuropathies by promoting nerve regeneration [42]. The final step of the reconstructive ladder would also be a final frontier in iPSCs; this is the full regeneration of a vascularized composite tissue such as a limb, custom-grown from a patient’s cells. Pre-clinical efforts to begin vascularized composite allotransplantation (VCA) regeneration are discussed in Section 3.2.7.

At present, iPSCs in plastic and reconstructive surgery are most often combined with a biomaterial to either facilitate the growth of cells in a 3-dimensional fashion or to improve the engraftment of cells [43,44]. It is important to consider both the advantages and disadvantages of introducing a biomaterial. Biomaterials have the capacity to be conjugated with various growth factors that may promote the growth and maturation of iPSCs [44,45]. Similarly, the use of a biomaterial promotes the creation of 3-dimensional cell growth environments, giving further control over cell behavior, motility, and morphology [46]. The disadvantages of biomaterial scaffolding for iPSC growth include monitoring the interaction between cells and the biomaterial, as well as additional considerations regarding how biomaterial stiffness, shape, surface chemistry, and size can influence cell behavior, necessitating a thorough investigation ensuring the beneficial effects of biomaterials [44].

Another significant consideration regarding the cell environment is that of mono-culture versus co-culture when using iPSCs. Both mono-culture and co-culture are currently being actively investigated within the field of plastic surgery. Haubner et al. co-cultured adipose-tissue-derived stem cells with human fibroblasts, showing better cell proliferation after external radiation compared to either mono-culture [47]. Kim et al. successfully derived a skin organoid using a co-culture of iPSC-derived keratinocytes and fibroblasts [48]. Given the direction of iPSC use to produce composite tissues, it is likely that the benefits of co-culture will need to be explored in greater depth as the field progresses.

### 3.1. hiPSCs in Wound Healing Promotion

Plastic surgeons often play a crucial role in optimizing and managing wound healing, performing reconstructive surgery to replace tissue and restore form and function. Wound healing describes the body’s physiological response to a disruption. Wound healing is broken down into three phases: inflammatory, proliferative, and remodeling [49,50,51]. In brief, the inflammatory phase is marked by the formation of a platelet plug to prevent the exsanguination and chemotaxis of neutrophils and macrophages [49,50]. The proliferation phase includes the migration of fibroblasts activated by macrophages, subsequent type III collagen synthesis, angiogenesis, and re-epithelialization [49,50]. Lastly, in the remodeling phase, type III collagen is replaced by type I collagen to achieve a 4:1 ratio, and contraction is mediated by myofibroblasts. The last phase includes scarring and scar remodeling. This healing process can be delayed due to local factors such as infection, necrosis, pressure, poor blood supply and oxygenation, and debris, or systemic factors such as diabetes, malnutrition, immunosuppression, and smoking [51]. Given the wide scope of factors that can dysregulate the wound healing process, there is a significant wound healing failure rate and associated chronic wound burden [51].

iPSCs have been shown to have applications in enhancing each phase of wound healing. In the inflammatory phase, iPSC-derived cells can secrete growth factors and cytokines. This is of particular interest in diabetic patients who have suppressed cytokine secretion [33,52]. iPSC-derived cells secreting cytokines leads to increased macrophage migration, as well as the upregulation of angiogenesis [33]. Lu et al. demonstrated in a rhesus monkey model that both autologous and allogeneic iPSCs and their exosomes promoted accelerated wound healing when applied topically to a wound, while autologous iPSCs were more effective [53]. A major limitation of iPSC use in clinical practice is the formation of benign teratomas due to the host immune response. Lu et al. also noted that all autologous iPSCs formed teratomas compared to allogeneic iPSCs [54]. Gorecka et al. demonstrated that hiPSC-derived smooth muscle cells embedded in collagen scaffolds applied to a diabetic mouse wound model led to accelerated wound healing due to increased angiogenesis, compared to collagen scaffolding alone [34]. Shen et al. demonstrated in a T1DM mouse model that iPSC-derived endothelial progenitor cells transplanted into full-thickness wounds resulted in accelerated wound closure [55]. Clayton et al. similarly demonstrated in an immunodeficient mouse model that the transplantation of iPSC-derived endothelial cells into full-thickness wounds led to expedited wound closure due to increased wound perfusion [56].

### 3.2. hiPSCs in Reconstructive Tissue Regeneration

Skin grafting and flaps represent options for wound closure and require the procurement and transfer of autologous tissue from a donor site [3,4]. Donor site availability is of concern in patients with significant tissue defects such as extensive burns, or in patients who require repeated reconstruction due to the failure of prior reconstructive efforts [3,4,5,6,7,57]. Donor site morbidity also poses a significant challenge to a patient’s quality of life and satisfaction following their reconstruction [8]. The use of hiPSCs to grow autologous skin grafts and flaps in the future may circumvent donor site availability and morbidity, ushering in a new era of regenerative medicine in plastic surgery. A summary of literature on hiPSC use in various tissue regeneration is shown in Table 1.

#### 3.2.1. Skin

Currently, the mouse has been the standard for investigating skin diseases and developmental mechanisms in vivo. However, the possibility of creating a personalized model for testing treatments—using autologous rather than xenogenic cells for future applications—circumvents concerns regarding the translation of research to human clinical use. Through the application of differentiation protocols, hiPSCs can be differentiated into somatic cells, herein referred to as iPSC-derived cells, to create three-dimensional skin organoids. Bilousova et al. first reported iPSCs differentiated into a keratinocyte lineage that displayed the characteristics of skin and appendages [58]. Lee et al. describe a 130-day in vitro protocol that resulted in an organoid with multiple complex skin cell layers, including hair follicles, adipocytes, melanocytes, sebaceous glands and sensory neurons [59]. These organoids mimic the structural and functional characteristics of native skin, comprising multiple cell layers. Skin organoids could be applied to large skin defects that require reconstruction by creating a readily available skin source for reconstruction. Most current skin substitutes do not replicate the key appendages of the skin [60]. hiPSCs derived from cord blood mononuclear cells have been used to differentiate into keratinocytes and fibroblasts to generate epidermal and dermal layers, respectively. These were combined atop each other to generate a 3D skin organoid [48]. The subsequent transplantation of this human skin organoid into immunodeficient mice demonstrated wound-healing ability, revealing a similar morphology to real skin 2 weeks after transplantation [48]. Lee et al. also performed a transplantation of their hiPSC-derived skin organoids into mice and found that after 1 month, the organoids had integrated into the host skin with outward-growing hair [59]. This demonstrates a potential avenue for providing a scalable and accessible source of skin grafts for reconstruction.

#### 3.2.2. Vasculature

Perfusion is a critical consideration in plastic surgery, as the survival of transplanted tissue hinges on appropriate arterial inflow and venous outflow. hiPSCs have been shown to have the ability to derive endothelial cells, which can in turn form functional blood vessels. Kusuma et al. showed that hiPSCs were capable of self-organizing into a vascular scaffold, which could be transplanted into a mouse model and integrated into the host vasculature [61]. Rosa et al. differentiated hiPSCs into arterial- and venous-like endothelial cells (ECs), and determined that they were responsive to pro-inflammatory markers, though slightly less than somatic ECs, and responded to vasoactive agonists the same as somatic ECs [62]. Lin et al. describe that a challenge with hiPSC-derived EC is that they at times lack certain arterial or venous markers, whilst Olmer et al. and Halaidych et al. describe that some markers are co-expressed [63,64,65]. Samuel et al. was able to generate functional blood vessels from hiPSCs that were grafted in a mouse model for 280 days successfully [66]. The generation of de novo vasculature from hiPSCs offers a potential for mitigating the avascular nature of other hiPSC-derived tissue.

#### 3.2.3. Nerve

Restoring sensorimotor function to tissue in the context of wound healing and reconstruction is another key objective of plastic surgery. In mice with sciatic nerve division, Ikeda et al. reported enhanced peripheral nerve regeneration and the recovery of motor function using bioabsorbable nerve conduits combined with support iPSCs [67]. Another study by this same group used tissue-engineered nerve conduits enhanced with a 3D-culture of iPSC-derived neurospheres for the treatment of peripheral nerve defects [68]. Schwann cells, which support axonal growth and myelination in the periphery, are known to be crucial in peripheral nerve healing and regeneration [69]. Sourcing these cells in sufficient quantity for human use, however, has proven difficult. Several protocols for differentiating iPSCs into neural crest cells and subsequently Schwann cells have been described [70,71,72]. More work is needed to understand how different protocols for iPSC-derived Schwann cells perform in the regeneration of peripheral nerves, in comparison to each other as well as the current standard of care: autografting [69]. In addition to its prospective use as a peripheral nerve treatment modality, Mittal et al. describe the current use of iPSCs in the disease modeling of peripheral neuropathies [42]. Malheiro et al., for example, report the use of hiPSC-derived nociceptors for peripheral nerve modeling and tissue reinnervation strategies [73]. Impressively, the nociceptors demonstrated electrical activity and responsiveness to noxious stimuli. Recently, Powell and Philips described 3D cultures of hiPSC-derived Schwann cells for the in vitro regeneration of peripheral nerves [74].

#### 3.2.4. Skeletal Muscle

Skeletal muscle regeneration poses challenges due to the limited regenerative capacity of mature muscle tissue, leading to the formation of fibrotic scar tissue and compromised functional recovery after injury or degeneration. The scarcity of resident stem cells within skeletal muscle further exacerbates this challenge. However, iPSCs offer a promising solution. iPSCs can be directed to generate myogenic progenitors that facilitate efficient muscle regeneration or used for disease modeling. Osaki et al. used iPSCs differentiated into functional muscle cells and engineered optogenetic motor neurons to respond to light stimulation to 3D model amyotrophic lateral sclerosis (ALS). They were able to use this ALS on-a-chip model to investigate the pathogenesis and analyze possible drugs for the treatment of the condition [75].

#### 3.2.5. Cartilage

Cartilage has a limited capacity for intrinsic healing. Unlike some tissues in the body, cartilage lacks robust regenerative mechanisms, making it less capable of repairing damage caused by injury or degeneration. Nguyen et al. [76] reported the use of hiPSCs in cartilage differentiation through 3D bioprinting using iPSCs, a nanofibrillated cellulose bioink, and irradiated human chondrocytes [76]. Nakamura et al. were able to successfully fabricate a scaffold-free homogenous articular cartilage construct using hiPSC-derived neural crest cells that were differentiated into chondrocytes over a five-week maturation period [77]. In another study by Choi et al., hiPSC-derived chondrocytes were used to fabricate cartilaginous extracellular matrix, overcoming primary chondrocyte tendencies to take on a more quiescent and fibrocartilaginous phenotype in vitro [78]. The decellularized matrix generated from the hiPSC-derived chondrocytes demonstrated the enhanced in vitro chondrogenesis of iPSCs when recellularized, and also showed the enhancement of osteochondral defects in rats compared to controls. This unveils the potential for the treatment of larger defects using customizable bioprinted cartilage and the reconstructive surgery of cartilaginous sites such as the ears, nose, and ribs. In the future, we may see heparan sulfate proteoglycans such as perlecan combined with iPSCs, as they have demonstrated success in the regeneration of cartilage by harboring growth factors and therefore promoting neovascularization [79]. Cotreatment using iPSCs and these proteoglycans may potentiate their effects on cartilage regeneration, leading to better treatment outcomes.

#### 3.2.6. Bone

Bone regeneration using iPSC holds significant importance in the field of regenerative medicine for plastic and orthopedic surgery. While most fractures typically heal with no complications, large skeletal bone defects require surgical intervention. Autologous bone grafting is the gold standard for treatment; however, iPSC-based bone regeneration provides an alternative that eliminates the need for additional surgical procedures and the potential complications associated with graft harvesting. Kang et al. demonstrated the first differentiation of hiPSCs into functional osteoblasts using adenosine without teratoma formation [80]. iPSC-derived MSCs have been reported to improve bone regeneration in animal models [81]. Qi et al. used hiPSC-derived MSCs on osteoporotic rats to repair calvarial defects, demonstrating that exosomes derived from hiPSC-MSCs exert a regenerative impact on cutaneous wound healing by promoting angiogenesis and osteogenesis [82].

**Table 1:** Summary of the literature describing iPSC-derived tissue regeneration.

Tissue Type	Study	Model Organism	Type of iPSC	Major Findings
Skin	Bilousova et al., 2011 [58]	Mouse	iPSC-derived keratinocytes	iPSCs can be derived into functional keratinocytes with similar characteristics to primary keratinocytes. They show s the potential to produce epidermis, hair follicles, and sebaceous glands in vivo.
	Lee et al., 2022 [59]	In vitro	hiPSC-derived skin organoids	hiPSCs can be derived into skin organoids, which, after 60 days of incubation, produce hair follicles and, after 130 days, have stratified skin layers, pigmented hair follicles, and glands.
Vasculature	Kusuma et al., 2013 [61]	Mouse	hiPSC-derived early vascular cells	Early vascular cells are able to differentiate into endothelial cells and pericytes, which can self-organize into microvascular networks on a scaffold, and can survive and integrate into the host vasculature.
	Samuel et al., 2013 [66]	Mouse	hiPSC-derived endothelial cells	hiPSCs can generate endothelial cells, which then form blood vessels that can last 280 days in vivo. hiPSCs can also be used to derive endothelial cells and form blood vessels in vivo.
Nerve	Ikeda et al., 2014 [67]	Mouse	iPSC-derived neurospheres	Sciatic nerve gaps could be filled through peripheral nerve regeneration and are fastest with the use of iPSC-derived neurospheres with reconstruction.
	Kim et al., 2017 [70]	In vitro	hiPSC-derived Schwann cells	hiPSCs can be derived into Schwann cell precursors and can functionally secrete neurotrophic factors and myelination potential in vitro and in vivo.
	Liu et al., 2012 [71]	Chicken embryo	hiPSC-derived neural crest cells	hiPSCs can be induced to produce neural crest stem cells, which exhibit similar characteristics to endogenous embryonic neural crest cells. This is also the first report of myelination by hiPSC-derived Schwann cells.
	Huang et al., 2017 [72]	Rat	hiPSC-derived neural crest stem cells and Schwann cells	hiPSC-derived cells can be used to construct a nerve conduit and implanted into a rat sciatic nerve transection model, with significantly higher electrophysiological recovery at 1 month than the acellular group.
	Malheiro et al., 2021 [73]	In vitro	hiPSC-derived nociceptors	hiPSCs can be differentiated into nociceptors and used for peripheral nerve modeling and tissue reinnervation strategies.
Skeletal Muscle	Osaki et al., 2018 [75]	In vitro	hiPSC-derived muscle cells and optogenetic motor neurons	iPSCs differentiated into functional muscle cells and optogenetic motor neurons can be engineered to respond to light stimulation to 3D model ALS.
Cartilage	Nguyen et al., 2017 [76]	In vitro	hiPSC-derived	hiPSCs can be used in 3D bioprinting using a nanofibrillated cellulose bioink and irradiated human chondrocytes for cartilage regeneration.
	Nakamura et al., 2021 [77]	In vitro	hiPSC-derived chondrocytes	hiPSC-derived chondrocytes can be used to create cartilage constructs up to 6 cm^2^ using bio-3D printing.
	Choi et al., 2023 [78]	Rat	hiPSC-derived chondrocytes	Decellularized hiPSC-derived chondrocytes demonstrate enhanced in vitro chondrogenesis when recellularized, and show the enhancement of osteochondral defects in rats.
Bone	Kang et al., 2016 [80]	Mouse	hiPSC-derived osteoblasts	hiPSCs contribute to the restoration of critical-sized bone defects by generating neobone tissue without the occurrence of teratoma formation.
	Qi et al., 2016 [82]	Rat	hiPSC-derived MSCs	Exosomes derived from hiPSC-MSCs exert a regenerative impact on cutaneous wound healing by promoting angiogenesis and osteogenesis.

#### 3.2.7. Vascularized Composite Allotransplantation

Vascularized composite allotransplantation is an emerging reconstructive option wherein multiple tissue subunits are transplanted as a unit. Hand and face transplants have only emerged within the past 25 years and are the highest complexity reconstructive options for patients with a significant loss of form and function. Donor availability that is appropriately typed and matched to the recipient with the possibility of rejection are significant concerns. The warm ischemia time refers to the timeframe wherein ischemic tissue will sustain permanent damage, in the absence of any cooling. An added complication in VCA is that skeletal muscle has a warm ischemia time of only 3–4 h [83]. In parallel to VCA, the most sophisticated application of iPSC would be one that presents a solution to the availability, immunogenicity, and preservation of limbs. Research has started moving in this direction in the regeneration of distal appendages. Lin et al. focused their research on Xenopus frogs, as some amphibians are capable of limb regeneration after amputation [36]. Lin et al. was able to show that endogenous limb progenitors enhanced frog limb regeneration and anticipated iPSC use in grafting limb amputation sites [36]. Notably, Lin et al. found that cells were delivered most optimally with a fibrin patch [36]. Chen et al. have demonstrated that limb progenitor-like cells can be derived from iPSCs [37]. This group showed that these iPSC-derived cells express appropriate genes and, when transplanted within a fibrin matrix biomaterial for engraftment, promote phalange regeneration in a mouse model [37].

Human fingertips distal to the nail bed have the capacity to regenerate. Findings by Chen et al. suggest that future directions include the regeneration of more proximal levels [37]. Mori et al. successfully showed that murine iPSCs were capable of producing a limb-bud mesenchyme with the potential to develop into a limb [84]. Their group also demonstrated that limb-bud mesenchyme can contribute to the development of a limb through transplantation studies [84]. Yamada et al. showed that hiPSC-derived limb-bud mesenchymal cells can be used to form hyaline cartilage-like tissue [85]. This work offers a start to studying human skeletal derivation and provides a new cartilage model for drug testing [85]. To date, we are unaware of any studies that have been able to grow in vitro human appendages to be used for disease modeling and functional transplantation.

### 3.3. hiPSC in Pathology of the Skin

Animal models have been used historically for the evaluation of skin form and function. hiPSCs represent a promising new model. Rodent models are limited in that their eccrine sweat glands, sebaceous glands, and hair follicles have differing compositions and densities compared to human morphology [86,87]. hiPSCs can differentiate into skin keratinocytes, fibroblasts, hair follicles and sebaceous glands. hiPSC-derived organoids have been used in certain skin conditions such as epidermolysis bullosa and scleroderma [88,89]. Epidermolysis bullosa is a blistering disease that affects 1 in 30,000 people and is caused by the loss of structural integrity in the epidermal–dermal junction (EDJ), often due to genetic mutations (e.g., COL7A1 for collagen VII). Ramovs et al. characterized the EDJ within hiPSC-derived skin and determined that they were able to generate hair-bearing skin organoids with a protocol described by Lee et al. [59,88]. Interestingly, the EDJ lacked collagen VII, which anchors the epidermis to dermis, creating a model that may replace the need for animal models in studying epidermolysis bullosa [88]. Localized scleroderma is a connective tissue disease characterized by the atrophy of sweat glands, bullae, and necrotic keratinocytes at the EDJ. Ma et al. developed a model for localized scleroderma using iPSC-derived epidermal and mesenchymal organoids, which were then grafted into mice [89]. Ma et al. demonstrated a new application of iPSCs as a disease model for scleroderma and also offered a regenerative medicine solution [89].

Skin cancer management is a significant portion of plastic surgery practice, and excision with appropriate margins can lead to skin defects requiring skin grafting or more sophisticated wound closure. In the future, iPSC-derived skin organoids may serve as a tissue replacement in cases where donor site availability or morbidity is of significant concern. iPSCs have also been used to model skin cancer for research regarding its mechanisms of recurrence and for drug testing. Castro-Pérez et al. produced iPSCs by reprogramming melanocytes to study drug-resistant melanoma [90]. Interestingly, they determined that advanced melanoma exhibits heightened resistance to iPSC reprogramming, signifying a loss in plasticity [90]. They hypothesized that their strategy of reprogramming melanoma may present a model for studying melanoma that is aggressive and drug resistant [90]. iPSCs have also been investigated as a possible avenue for treating melanoma. Wu et al. found that interleukin-24 (IL-24), which is a gene that can induce the apoptosis of melanoma cells, integrated into hiPSCs can inhibit melanoma growth in mice [91]. iPSCs have also been implicated in nevoid basal cell carcinoma syndrome (NBCCS), a rare autosomal dominant disease. Navarro et al. have found that it is particularly important to capitalize upon iPSCs as a model for evaluating childhood malignancies such as NBCCS, as these are often caused by germline mutations [92]. Ikemoto et al. were able to derive iPSCs with a specific mutation from a family with NBCCS and believe that, in the future, it will be a very powerful tool for drug testing and investigating this rare syndrome [93]. Squamous cell carcinoma (SCC) has also been modeled through iPSCs successfully. Koh et al. were successful in identifying iPSC markers in cancer stem cells within head and neck SCC, offering a target for new therapies [94]. Verusingam et al. were able to reprogram oral SCC into iPSCs in order to use them in future studies for anti-cancer therapy testing [95]. Rami et al. took a special interest in cutaneous SCC in individuals with recessive dystrophic epidermolysis bullosa [96]. They were able to reprogram these SCC cells into iPSCs successfully to create a tool for studying SCC in this context [96]. Overall, significant efforts have been made to create iPSC models for BCC, SCC, and melanoma, all of which are skin cancers that are often managed by plastic surgery. A summary of disease modeling using iPSCs is provided in Table 2.

## 4. Discussion

### 4.1. Challenges and Limitations of hiPSC in Disease Modeling

A hope in the future of hiPSC disease modeling is the capacity to decrease and ultimately eliminate the use of animal models. It is particularly appealing to consider using hiPSC modeling as animal models frequently fail drug testing due to different biological responses in comparison to humans. However, hiPSC-based disease modeling does present limitations. An inherent limitation of hiPSCs is that their capacity to divide indefinitely provides an opportunity for chromosomal aberrations and genetic mutations. This may make in vitro hiPSCs non-representative of cells in vivo [97]. Another challenge of iPSC-based disease modeling is that they are much easier to create and validate for diseases of monogenic origin than polygenic complex diseases [97]. Next-generation sequencing platforms, large cohort lines, and gene editing may help tackle polygenic diseases. A limitation specific to iPSC-derived skin is vascularization, as most of the current organoid models lack blood vessels [59]. This would result in necrosis upon transplantation [59]. Kong et al. tried to tackle this issue by using an hiPSC-derived endothelial cell capillary network to circumvent flap morbidity by connecting it to a vascular pedicle in vivo [98].

### 4.2. Challenges and Limitations of hiPSC in Plastic Surgery

#### 4.2.1. Tumorigenicity and Off-Target Induction

Despite their advantages in tissue healing and reconstruction, iPSCs present their own host of challenges that currently limit clinical translation. It is known that the use of stem cells carries inherent risks of tumorigenesis, potentially leading to the in vivo development of teratocarcinomas or even somatic tumors [19,99,100]. Studies suggest that hiPSCs harbor a greater risk of tumor formation compared to hESCs. For example, chromosomal aberrations are more readily acquired in hiPSCs compared to ESCs, due to genetic and epigenetic factors from their somatic cell origin, reprogramming stress, and culture conditions [19]. Transfection protocols for the induction of stem cell characteristics often employ genes that are highly expressed in various cancers. These protocols thus offer the possibility of gene-reactivation in situ, potentially leading to de-differentiation and subsequent tumorigenesis.

In an effort to reduce the tumorigenic potential, new pluripotency induction techniques have been designed to circumvent the use of the transcription factors Oct4/Sox2/c-Myc/KLF4, including protocols that forego the use of Myc [18,101], or use a transformation-deficient Myc variant [102]. Later studies found that the inhibition of tumor suppressor gene p53 enables reprogramming with just two factors, Oct4 and Sox2 [103]. Reprogramming methods that overcome the tumorigenic risks associated with genetic alterations from viral integration have also been described, including synthetic modified mRNAs, the direct delivery of reprogramming proteins, and piggyBac transposition [104,105,106,107,108,109,110,111,112]. In more recent years, advanced gene-editing methodologies have been designed and show promise in the generation of hiPSCs for disease modeling and other applications, including Zinc Finger Nucleases (ZFNs), Transfection Activator-Like Effector Nucleases (TALENs) and, of course, Clustered Regularly Interspaced Short Palindromic Repeats (CRISPRs)/cas9 [113].

hiPSCs are known to possess epigenetic memory from their somatic origin [114,115]. This has led to questions regarding whether certain cell types are safer sources of hiPSCs from a tumorigenic perspective. For instance, it has been posited that it may be safer to derive iPSCs from embryonic tissues such as cord blood, as they require the transduction of fewer factors to achieve pluripotency [116].

Beyond tumorigenesis, the use of hiPSCs presents the risk of off-target cellular differentiation and the growth of undesired cell or tissue types. For example, in their work on the development of iPSC-derived skin organoids for disease modeling and tissue regeneration, Lee et al. describe the aberrant proliferation of chondrocytes and myocytes [59].

In view of these challenges, a safer approach would be to develop treatments that employ a population of purely differentiated hiPSCs prior to use. Attempts have been made to accomplish this, including the use of cytotoxic antibodies against undifferentiated cells [117] and magnetism/fluorescence-based cell sorting [118]. Other strategies, such as incorporating drug susceptibilities into ESCs, have also been explored [119]. Still, achieving this level of differentiation efficiency remains challenging.

#### 4.2.2. Immunogenicity

It is known that iPSCs may still be subject to immune rejection, despite their (autologous) derivation from host cells [120,121,122,123]. Immunogenicity can be acquired over the process of somatic cell reprogramming, iPSC expansion, and differentiation into terminal cell types. These steps create vulnerable cellular states wherein de novo mutations can accrue, resulting in the expression of neoantigens [124]. The reprogramming process is estimated to cause a ninefold relative increase in mutation rate in culture [125]. Mitochondrial DNA (mtDNA) is even more susceptible to de novo mutations (10–20 times more than nuclear DNA) [126,127,128]. Even single-nucleotide polymorphisms in mtDNA are capable of causing immunogenic neoantigen expression [129].

The mechanism of iPSC immunogenicity is not fully understood. For ethical reasons, iPSC immunogenicity has yet to be sufficiently characterized in humans. Araki et al. showed that in inbred, syngeneic mice, iPSC-derived cardiomyocytes are highly immunogenic when transplanted from one B6 mouse to another [130]. Notably, the same study showed that iPSC-derived skin tissue cells obtained by injecting B6 iPSCs into B6 blastocysts showed immune tolerance. They proceed to suggest that not only do iPSCs exhibit similar levels of immunogenicity as ESCs, but also that clonal variations between terminally differentiated iPSCs exhibit differential immunogenicity. Guha et al. reported contrary results, showing that iPSC-derived cell populations representing all three germ layers could be engrafted into syngeneic recipient mice without immune rejection [131]. Studies in primates showed minimal immune response to iPSC-derived neural cells transplanted in the brain [132]. The validity of these results is limited by the suitability of some assays for assessing immunogenicity [130], and it has been found that even iPSCs expected to elicit an immune response have demonstrated immune tolerance in previous models.

The differentiation of iPSCs into mature phenotypes before clinical use may also be important from an immunologic standpoint. Immature cell types show increased immunogenicity that diminishes with differentiation, owing to both their relative underexpression of MHC Class-I and their overexpression of embryonic/fetal proteins [133]. Low MHC Class I levels subject cells to natural killer cell attack, threatening engraftment. The expression of embryonic or fetal proteins, as seen in some cancers, presents an antigenic target to the host immune system. Thus, it is important that iPSCs are adequately differentiated into target tissue types to avoid rejection. Substantial progress has been made in the differentiation of iPSCs and hiPSCs into adipocytes [134] and keratinocytes [58,135].

Gene-editing strategies such as CRISPR/cas9 may also be able to address issues of immunogenicity by introducing specific genes into the safe harbor loci that can confer immune protection to hiPSCs, as has previously been explored with hESCs [113,136].

Although preliminary evidence is conflicting, concerns of hiPSC immunogenicity may not limit their clinical translation in reconstructive surgery. More work must be done to elucidate the potential associations between iPSC immunogenicity and factors such as epigenetic memory, pluripotency induction techniques, differentiation protocols, and recipient site characteristics. The modification of variables such as the somatic origin of iPSCs and gene editing for particular applications may also mitigate immunogenicity.

#### 4.2.3. Pragmatism

In the foreseeable future of clinically translatable hiPSC-based disease models and treatments in reconstructive surgery, a more practical challenge presents itself. The nature of disease modeling and drug testing necessitates the reproducibility of results, which is only possible in a setting where disease models can be generated in sufficient numbers and with reasonable homogeneity. hiPSC generation and differentiation is an undoubtedly costly process, which may present barriers to extensive disease research using this approach. As discussed earlier, tumorigenicity and off-target induction may threaten our ability to generate such homogeneity in disease models. On the other hand, hiPSC-based disease models may find their niche as a preliminary screening measure for drug and treatment testing, before moving into animal or human trials.

From a clinical perspective, the attractiveness of hiPSCs in reconstructive surgery is the possible generation of genetically identical tissue sources for patients who lack viable donor sites. These patients are vitally unstable and require active management that is not amenable to somatic cell collection, pluripotency induction, differentiation, and tissue engineering. In these cases, the risk of delaying treatment to procure cells, re-program them into hiPSCs, and grow the appropriate tissue may be too high. Lee et al. state that their protocol of forming skin organoids with appropriate appendages is a meticulous process that takes 4–5 months [59]. The process is also noted to be quite labor intensive [59]. At present, it is possible that we may see the practical application of skin organoids for chronic wound coverage. However, in instances where we most require exogenous tissue coverage, typically in the acute setting, the field requires significant advances to accelerate the process to allow for clinical use.

## 5. Conclusions and Future Directions

The work performed on iPSCs has given tremendous insight into the potential uses of hiPSC-based therapies in regenerative medicine, presenting a new and exciting approach to plastic surgery practice. Over the past two decades, substantial progress in iPSC-based disease modeling, wound healing enhancement, graft/flap engineering, and tissue regeneration has been made. Skin organoids constructed using iPSCs have demonstrated their use in modeling various diseases commonly addressed in plastic surgery practice, including epidermolysis bullosa, scleroderma, SCC, BCC, and melanoma. The simulation of these diseases has profound implications for our ability to research and develop corresponding medical and surgical treatment. Disease modeling may potentiate personalized solutions to diseases such as drug-resistant or refractory skin cancers. Moreover, future work in these areas may permit the modeling of a greater number of disease states that are beyond the scope of plastic surgery but incredibly impactful to medicine as a whole.

iPSCs have demonstrated their significant use as potentiators of the healing process. iPSC injections into wound beds may enter clinical practice to promote the healing of severe wounds. Plastic surgeons may one day find the use of iPSCs integral to the treatment of wounds, especially in the context of diseases that compromise wound healing such as diabetes. iPSCs have also shown success in their growth of skin, skin constituents, vasculature, nerves, cartilage, bone and skeletal muscle for use as additional tissue in tissue regeneration and reconstruction. In the future, differentiation techniques may lead to the tandem development of several tissue subunits, permitting the regeneration of vascularized composite tissues that circumvent the need for donor tissue.

Several challenges impede the clinical translation of iPSCs and hiPSCs in their current state. We have discussed tumorigenic, immunogenic, and pragmatic barriers to the adoption of iPSCs in human trials. In addition, epigenetic memory presents a complex variable in the development of iPSC-related therapies. A deeper understanding of how epigenetic memory influences the phenotypic properties of iPSC-derived disease models, treatments, tumorigenicity and immunogenicity will be necessary in order to take full advantage of this promising technology. New studies continue to characterize and devise solutions to these challenges, and there is no doubt that hiPSCs will one day play a role in the clinical setting of plastic surgery and beyond.

## Figures and Tables

**Table 2:** Summary of iPSC literature in skin pathology modeling.

Skin Pathology	Study	Model Organism	Type of iPSC	Major Findings
Epidermolysis Bullosa (EB)	Ramovs et al., 2022 [88]	In vitro	hiPSC-derived skin organoids	EDJ of skin organoid lacked collagen VII; gene mutations in COL7A1 producing collagen VII are common in EB.
Scleroderma	Ma et al., 2022 [89]	Mouse	hiPSC-derived epithelial and mesenchymal (EM) organoids	EM organoids can regenerate integral components of skin including sweat glands and blood vessels in the scleroderma skin model.
Basal Cell Carcinoma (BCC)	Ikemoto et al., 2017 [93]	In vitro	NBCCS-iPSCs	Examining the genetic makeup of iPSC clones helped in identifying mosaicism.
Squamous Cell Carcinoma (SCC)	Verusingam et al., 2017 [95]	In vitro	hiPSCs	Reprogrammed two cell OSCC cell lines (H103 and H376) into iPS-like cells; better maintenance of morphology and pluripotent expressions observed in Rep-H103 cells.
	Rami et al., 2021 [96]	Mouse	RDEB-cSCC-iPSCs	Reprogrammed and re-differentiated RDEB-cSCCs-iPSCs into keratinocytes showed reduced proliferative capacities in vitro and in vivo.
Melanoma	Castro-Pérez et al., 2019 [90]	Mouse	melanoma-derived iPSCs	Oncogenic BRAF inhibits melanocyte reprogramming. Melanoma-derived induced pluripotent stem cells (iPSCs) exhibit neural cell-like dysplasia and heightened resistance to MAPK inhibitors.
	Wu et al., 2020 [91]	Mouse	iPSC-derived MSCs	Interleukin-24 (IL-24) integrated into hiPSCs can inhibit melanoma growth.
